# Neuroinflammation in World Trade Center responders at midlife: A pilot study using [^18^F]-FEPPA PET imaging

**DOI:** 10.1016/j.bbih.2021.100287

**Published:** 2021-06-30

**Authors:** Yael Deri, Sean A.P. Clouston, Christine DeLorenzo, John D. Gardus, Elizabeth A. Bartlett, Stephanie Santiago-Michels, Lev Bangiyev, William C. Kreisl, Roman Kotov, Chuan Huang, Mark Slifstein, Ramin V. Parsey, Benjamin J. Luft

**Affiliations:** a Department of Medicine, Renaissance School of Medicine at Stony Brook University, Stony Brook, NY, USA; b Program in Public Health and Department of Family, Population, and Preventive Medicine, Renaissance School of Medicine at Stony Brook University, Stony Brook, NY, USA; c Department of Psychiatry, Renaissance School of Medicine at Stony Brook University, Stony Brook, NY, USA; d Department of Biomedical Engineering, Stony Brook University, Stony Brook, NY, USA; e Molecular Imaging and Neuropathology Area, New York State Psychiatric Institute, New York, NY, USA; f Department of Psychiatry, Columbia University Medical Center, New York, NY, USA; g Stony Brook World Trade Center Wellness Program, Renaissance School of Medicine at Stony Brook University, Stony Brook, NY, USA; h Department of Radiology, Renaissance School of Medicine at Stony Brook University, Stony Brook, NY, USA; i Taub Institute for Research on Alzheimer's Disease and the Aging Brain, Columbia University, New York, NY, USA

**Keywords:** World trade center, Neuroinflammation, Glial activation, Positron emission tomography, Translocator protein 18-kDa, Posttraumatic stress disorder, WTC, World Trade Center, MCI, Mild Cognitive Impairment, MoCA, Montreal Cognitive Assessment, HAB, high affinity binders, LAB, low affinity binders, MAB, mixed affinity binders, TSPO, Translocator protein 18-kDa

## Abstract

**Background:**

Neuroinflammation has long been theorized to arise from exposures to fine particulate matter and to be modulated when individuals experience chronic stress, both of which are also though to cause cognitive decline in part as a result of neuroinflammation.

**Objectives:**

Hypothesizing that neuroinflammation might be linked to experiences at the World Trade Center (WTC) events, this study explored associations between glial activation and neuropsychological measures including post-traumatic stress disorder (PTSD) symptom severity and WTC exposure duration.

**Methods:**

Translocator protein 18-kDa (TSPO) is overexpressed by activated glial cells, predominantly microglia and astrocytes, making TSPO distribution a putative biomarker for neuroinflammation. Twenty WTC responders completed neuropsychological assessments and *in vivo* PET brain scan with [^18^F]-FEPPA. Generalized linear modeling was used to test associations between PTSD, and WTC exposure duratiioni as the predictor and both global and regional [^18^F]-FEPPA total distribution volumes as the outcomes.

**Result:**

Responders were 56.0 ​± ​4.7 years-old, and 75% were police officers on 9/11/2001, and all had at least a high school education. Higher PTSD symptom severity was associated with global and regional elevations in [^18^F]-FEPPA binding predominantly in the hippocampus (*d* ​= ​0.72, *P* ​= ​0.001) and frontal cortex (*d* ​= ​0.64, *P* ​= ​0.004). Longer exposure duration to WTC sites was associated with higher [^18^F]-FEPPA binding in the parietal cortex.

**Conclusion:**

Findings from this study of WTC responders at midlife suggest that glial activation is associated with PTSD symptoms, and WTC exposure duration. Future investigation is needed to understand the important role of neuroinflammation in highly exposed WTC responders.

## Highlights

• We examined the theory that glial activation is associated with 9/11 exposures.
• TSPO-Vt was examined using PET in 20 responders adjusting for TSPO genotype.
• Responders with PTSD had increased TSPO distribution volume in the hippocampus.
• Heavily exposed responders had increased TSPO distribution in the parietal cortex.

## 1 Introduction

During the World Trade Center (WTC) attacks on 9/11/2001 and in their aftermath, the responders who aided in the search, rescue, and recovery efforts endured multiple traumatic and toxic environmental exposures (Dasaro et al., 2017). The men and women who participated in search, rescue, and recovery operations on the event were exposed to severe physical exposures and stressful experiences (Galea et al., 2002). Posttraumatic stress disorder (PTSD) has long been noted as one central feature of life after WTC exposures, with up to one-fifth of responders having significant and chronic indications of PTSD more than a decade after the events (Bromet et al., 2016).

WTC exposures may have caused neuroinflammation in responders. Indeed, recent work has pointed out that associations between higher levels of C-reactive protein, a marker of systemic inflammation and PTSD symptomatology have been identified in a large sample of residents and survivors exposed to the WTC disaster, with focal results among individuals reporting higher re-experiencing symptomatology (Rosen et al., 2017). Gene expression analyses have noted changes in mechanisms relating to inflammation in cell subpopulations focused on monocytes (Kuan et al., 2019) that were replicated and extended in follow-up analyses to explicate that monocyte activation was obvious in pathways linked to neural response to biotic and stressful exposures (Kuan et al., 2021). Further, targeted proteomic analyses have identified central markers of neuroinflammation and neurodegeneration in the blood of responders with PTSD (Kuan et al., 2020). Across this work, PTSD has consistently emerged as one mechanism through which neuroinflammatory processes might be engaged.

PTSD is a common outcome of exposure to a severely stressful event during which time individuals may have been exposed to death and injury or the threat thereof (APA, 2013). PTSD has been associated with increased risk of neuroinflammation has emerged as a potential biological mechanism in the pathogenesis and pathophysiology of PTSD (Hori and Kim, 2019). Meta-analytic evidence indicates a significant elevation of peripheral pro-inflammatory cytokines in individuals with PTSD suggesting that inflammation may play a role in promoting adaptive behavioral and physiological responses of PTSD (Passos et al., 2015). While such theories abound, the only prior positron emission tomography (PET) study investigating neuroinflammation in individuals with diagnosed PTSD reported that lower prefrontal limbic translocator protein 18-kDa (TSPO) binding was associated with greater PTSD symptom severity (Bhatt et al., 2020).

PET studies have predominantly demonstrated increased binding of TSPO, which is over-expressed in activated glial cells such as microglia and astrocytes (Bradburn et al., 2019, Knezevic and Mizrahi, 2018, Kreisl et al., 2018, Tournier et al., 2020b). Neuroinflammatory processes appear to play a crucial role in the progression from MCI to dementia (Chaney et al., 2019). Indeed, consistent with this interpretation, findings among WTC responders have identified increased PTSD symptom severity and prolonged exposures to the WTC sites are associcaed with increased risk of cognitive impairment (Clouston et al., 2016, Clouston et al., 2017, Clouston et al., 2019).

Recognizing that neuroinflammation is evident in many neurological and neurodegenerative conditions involving chronic exposures and cognitive dysfunction, the primary objectives of this pilot study were to investigate to what extent neuroinflammation was evident in WTC responders using *in vivo* PET brain scan. Specifically, we investigated whether global and/or regional [^18^F]-FEPPA binding was elevated in the brains of twenty WTC responders. As secondary analyses, we explored the possible link between [^18^F]-FEPPA binding and PTSD symptom severity and examined the relationship between [^18^F]-FEPPA binding and prolonged exposure to the WTC sites. Finally, we examined the association between [^18^F]-FEPPA binding and neurocognitive functioning.

## 2 Materials and methods

### 2.1 Study design and participants

The study was approved by the Stony Brook University Institutional Review Board (IRB# 1113241); approval for administration of radioactive ligand was given by the Institutional Radioactive Drug Research Committee (RDRC# 2017-018). All participants provided informed written consent at enrollment in the study and after all study procedures were fully explained.

Participants were recruited from a clinic-based monitoring program in the WTC Health Program (Dasaro et al., 2017) and who participated in an epidemiologic study of aging (Clouston et al., 2019).

*Inclusion Criteria*: All participants were between 45 and 65 years-old, had no evidence of dementia, had body mass index (BMI) ​≤ ​40 ​kg/m^2^, were fluent in English, and the capacity to provide informed consent.

*Exclusion criteria*: history of psychosis, substance-related or addictive disorders (determined from relevant modules within the SCID); diagnosis of major depressive disorder (MDD) within the last two years; history of serious head trauma, stroke or other neurological disorders; current use of cognitively-active medications (e.g., methylphenidate), anti-anticoagulants or anti-inflammatory drugs; serious medical condition such as renal failure and autoimmune disease; known history of hepatitis C; any contraindication for PET or MRI scanning (e.g., pace-maker, metallic implants, pregnancy, etc.); classified as low affinity binders for TSPO polymorphism (see section 2.4.5).

Study groups (CU *versus* MCI) were matched for age at scan (years), sex (male *versus* female), race (white *versus* black), ethnicity (Hispanic *versus* non-Hispanic), current educational attainment (high school, some college, and bachelor's degree), occupation before 9/11 (police *versus* other [e.g., non-traditional responders including, for example, construction workers and volunteers]), current body mass index (BMI; kg/m^2^), cognitive impairment status, and TSPO *rs6971* genotype (high *versus* mix affinity binders).

### 2.2 Assessment

#### 2.2.1 WTC environmental exposure

We have previously reported that cognitive impairment, longitudinal analyses of MCI incidence, and cognitive dysfunction across domains of memory and throughput are associated with longer duration of physical presence at the WTC sites (Clouston et al., 2017). In this study, we followed prior efforts by examining the possible association between the length of time worked on the WTC sites (expressed in months and collected at enrollment in the parent study) and measures of neuroinflammation.

#### 2.2.2 Posttraumatic stress disorder

Current (i.e., active in the past month) and past PTSD were diagnosed using the Structured Clinical Interview for the DSM-5 (SCID) (First, 2014), a semi-structured interview was administered by trained clinical interviewers. Severity of PTSD symptoms was assessed using total score of PTSD Checklist (PCL-5) (Blevins et al., 2015). PTSD symptom clusters measured using subscales were calculated using reported symptom severity in the SCID for the following symptom domains: re-experiencing, avoidance, negative cognitions and mood, and arousal (King et al., 1998). PTSD diagnosis was not an exclusion criterion.

### 2.3 Potential confounders and related variables

#### 2.3.1 Cognitive assessments

Because cognitive dysfunction is a common correlate of neuroinflammation, we examined the potential for neuroinflammation to also explain cognitive dysfunction in this population. MCI was defined as previously described (Clouston et al., 2019) in a manner consistent with the National Institute on Aging and the Alzheimer's Association (NIA-AA) criteria (Albert et al., 2011). The MoCA was used in diagnosis to measure global cognitive functioning (Nasreddine et al., 2005). Responders' medical histories were examined and responders with potential cognitively impairing medical conditions or using drugs with cognitive impairment as a possible side effect were excluded. Domain-specific cognitive performance was independently assessed using a validated computer-based battery (Maruff et al., 2009), which consists of repeated game-like tasks administered in a laptop-based format (Hammers et al., 2011; Lim et al., 2015; Racine et al., 2016). Six outcome measures were used to indicate cognitive function across domains of response time (Detection, answers per log_10_ ​ms), processing time (Identification, responses per log_10_ ​ms), visual memory (One-Card Learning, arcsine [proportion of correct responses^1/2^]), episodic memory (Continuous Paired Associate Learning, arcsine [proportion of correct responses^1/2^]), visuospatial learning (Groton Maze Learning Test, number of errors), and visuospatial memory (Groton Maze Learning Test Recall, number of errors).

#### 2.3.2 Major depressive disorder

Diagnosis of current (i.e., active in the past month) and past MDD were assessed using the Structured Clinical Interview for the DSM-5 (SCID-5) (First, 2014), a semi-structured interview administered by trained clinical interviewers. Although MDD diagnosis within two years of participation was exclusionary, we assessed depressive symptom severity using the Patient Health Questionnaire (PHQ-9) (Kroenke et al., 2010).

#### 2.3.3 Tobacco and alcohol use

Since both tobacco smoking and harmful alcohol use have been previously shown to be associated with neuroinflammation (Brody et al., 2017; Hillmer et al., 2017), both self-report tobacco smoking status (never, former *versus* current) and alcohol use (measured by the Alcohol Use Disorder Identification Test; AUDIT) were noted using monitoring data.

#### 2.3.4 TSPO rs6971 genotype

Blood samples were drawn to determined TSPO affinity genotype based on the rs6971 polymorphism within the TSPO gene (Ala147 → Thr147) prior to enrollment, at screening. All subjects were genetically profiled and classified as high affinity binders (HABs, Ala147/Ala147), mixed affinity binders (MABs, Ala147/Thr147) or low affinity binders (LABs, Thr147/Thr147) (Kreisl et al., 2013a; Owen et al., 2011). Subjects who were classified as LABs were excluded from the study based on previous work establishing the effect of TSPO *rs6971* genotype on affinity of [^18^F]-FEPPA for TSPO (Mizrahi et al., 2012).

### 2.4 Neuroimaging protocol

#### 2.4.1 Imaging acquisition

Brain [^18^F]-FEPPA PET scans were performed at the Stony Brook Ambulatory Care Pavilion using a 3T Siemens Biograph mMR hybrid imaging system (Siemens Healthineers, Knoxville, TN, USA). [^18^F]-FEPPA was synthesized in-house using a procedure modified from the literature (Wilson et al., 2008). Up to 185 MBq of [^18^F]-FEPPA was injected as an intravenous bolus, and continuous 3D dynamic PET were acquired in list-mode for 120 ​min following the radioligand injection as described previously (Rusjan et al., 2011).

For PET coregistration, region of interest (ROI) delineation, and partial volume correction (PVC) a three-dimensional T1-weighted structural magnetization-prepared rapid gradient echo (MPRAGE) structural MRI was obtained simultaneously to PET data acquisition with the following parameters: TR ​= ​2300 ​s, TE ​= ​3.24 ​ms, TI ​= ​900 ​ms, flip Angle ​= ​9°, acquisition matrix: 240 ​× ​256, voxel resolution: 0.87 mm^3^ isotropic, and duration of 5 ​min 40 ​s. All structural MRIs were read by a neuroradiologist for incidental findings.

#### 2.4.2 Input function measurement

An automated blood sampling system (Twilite; Swisstrace, Zurich, Switzerland) was used to continuously measure arterial blood radioactivity for the first 6 ​min of the scan. Additionally, seven discrete manual arterial blood samples were taken at 2.5, 7, 15, 30, 60, 90, and 120 ​min following injection, allowing whole blood and plasma radiotracer activity to be measured for input function determination to perform full kinetic modeling. Parent tracer and metabolite levels were measured using HPLC analysis for manual blood draws as previously described (Rusjan et al., 2011).

#### 2.4.3 Imaging processing and analysis

The PET images were reconstructed into a series of 20 timeframes according to the binning scheme: 1 ​× ​120, 2 ​× ​60, 3 ​× ​120, 6 ​× ​300, and 8 ​× ​600 ​s. Raw list-mode PET data was reconstructed offline using Siemens’ e7 Tools software and a CT-like MR-based attenuation map (Izquierdo-Garcia et al., 2014, Ladefoged et al., 2017). Motion correction and PET-to-MRI coregistration were done before time activity curves were generated as previously described (Iscan et al., 2017). To obtain a quantitative measure of [^18^F]-FEPPA uptake, two-tissue-compartmental (2 ​TC) kinetic modeling was performed to quantify the total distribution volume (*V*_T_) as the outcome measure, as previously validated (Rusjan et al., 2011). The whole blood to plasma ratio data was fit and then used to convert the whole blood automated early arterial sampling measurements to plasma. These early plasma estimates were then concatenated with the manual arterial plasma draws. The kinetic analysis for [^18^F]-FEPPA was performed using a Hill function to fit the percentage of unmetabolized tracer, and a straight line from time zero to the peak followed by the sum of three exponentials after the peak to fit the arterial input function. The arterial whole blood curve was used to correct for activity in the vascular component, assuming that the blood volume is 5% of the total brain volume (Leenders et al., 1990). [^18^F]-FEPPA *V*_T_ was calculated for each of the following ROIs: frontal cortex, parietal cortex, temporal cortex, occipital cortex, cingulate cortex, hippocampus, and whole brain.

Our previous work observed smaller hippocampal volume and reduced cortical thickness in WTC responders with cognitive impairment consistent with mild dementia (Clouston et al., 2020, Deri et al., 2021). Therefore, while dementia was an exclusion criterion, volumetric, and cortical thickness measures in the same ROIs were also used to test whether similar changes are evident in responders with MCI. The T1-weighted MPRAGE images were used to obtain volumetric and cortical thickness measures utilizing FreeSurfer (V.6.0; http://surfer.nmr.mgh.harvard.edu) on a Linux-based computing cluster. Cortical ROIs structural measures were extracted using the standard, automated cortical reconstruction pipeline (Dale et al., 1999; Fischl et al., 1999). Briefly, the surface models were inflated and registered to a spherical surface atlas, and cortical ROIs were parcellated according to the Desikan-Killiany atlas (Desikan et al., 2006). Additionally, volumes were also obtained for the hippocampus using FreeSurfer's automatic subcortical segmentation pipeline (Fischl et al., 2002). Bilateral volumetric measures were calculated as the sum of the unilateral ROI volumes and bilateral cortical thickness measures were calculated as a weighted average using the surface area of each hemisphere as the weighting factor. Estimated total intracranial volume (eTIV) was also extracted using FreeSurfer V.6.0. FreeSurfer outputs were visually inspected by overlaying the pial and white matter surface models onto the T1-weighted images and were reviewed for any inaccuracies. Minimal manual edits were necessary for these subjects. Images were then run through FreeSurfer a second time, beginning at the point where edits were applied. Hippocacmpal segmentations were visually inspected for inaccuracies; no data was excluded as a result.

### 2.5 Statistical analyses

Statistical analyses were conducted in RStudio V.1.2.1335 with R programming language V.3.5.2. Descriptive statistics were reported for the whole sample and separately for the subgroups of WTC responders. Results for continuous variables were reported throughout the text as mean and standard deviation (Mean ​± ​SD) or median and range (Median [Range]), depending on the normality of the variable's distribution.

Analyses examined the independent contributions of WTC exposures were examined using GLM model with both total PTSD symptom severity and length of time spent at WTC sites as predictors. Bivariate analyses for continuous variables relied on two-tailed independent-samples t-tests and nonparametric trend tests to calculate p-values. Results for categorical variables were reported as frequencies and percentages, and analyses relied on Fisher's exact tests to provide p-values. Multivariable-adjusted analyses examining group differences in global and regional [^18^F]-FEPPA *V*_T_ relied on generalized linear models (GLM) to fit a model using a Gamma distribution with a log link function for each ROI separately. Gamma distributions were used because the outcome, TSPO distribution volume, represents a count-like data generation process that often causes skewed data. Multivariable analyses included genotype status (MABs *versus* HABs) as a fixed effect to adjust for the effect of TSPO *rs6971* polymorphism on radioligand binding affinity. Additionally, multivariable-adjusted secondary analyses examined main effects of the associations between [^18^F]-FEPPA binding in each ROI and WTC exposure duration and PTSD symptom severity variables (i.e., PTSD severity total score and symptom clusters sub-scores), as well as domains of neurocognitive functioning. Coefficients (B), standard error for the beta coefficients (SE), correlation coefficients (r), and Cohen's *d* (*d*) were reported for regression models. Because secondary analyses were exploratory, both nominal differences (α ​= ​0.05), and differences that were statistically significant after accounting for the false discovery rate (FDR ​= ​0.05) (Benjamini and Hochberg, 1995) were reported in the text (*P* and *P*_FDR_ respectively). To ensure that potential differences in regional brain volumes due to potential neurodegeneration were not driving the results, partial volume correction (PVC) was conducted using region-based PVC with PETPVC toolbox (Thomas et al., 2016) and all regression models were repeated using the PVC [^18^F]-FEPPA *V*_T_ as the outcome variables. Finally, linear regression models examining volumetric and cortical thickness measures were performed for each ROI separately.

## 3 Results

### 3.1 Sample characteristics

Among 27 subjects screened, 23 met eligibility criteria but three withdrew from the study before undergoing PET brain scan yielding 20 WTC responders who completed study procedures (age range: 48–64 years).

Descriptive characteristics and PET parameters of the participants are presented in Table 1 for the whole sample and subdivided by cognitive status groups (CU *versus* MCI). This sample was mostly males, and most had at least some college education. As expected, there were no differences in any of the demographic characteristics used to match the groups including age, sex, race, ethnicity, educational attainment, occupation before 9/11 and BMI, and no difference in TSPO *rs6971* genotype distribution. Additionally, there was no significant difference between groups in smoking status or level of alcohol use. There were no significant group differences in PET parameters for [^18^F]-FEPPA including injected radioactivity dose and specific activity.

**Table 1:** Participant's demographics and PET parameters.

Characteristic	Overall (N ​= ​20)			
**Age at scan (years)**	56.00 ​± ​4.72			
**Age at 9/11 (years)**	39.35 ​± ​4.85			
**Sex**				
Male	18 (90%)			
Female	20 (10%)			
**Race**				
White	18 (90%)			
Black	20 (10%)			
**Ethnicity**				
Non-Hispanic	18 (90%)			
Hispanic	20 (10%)			
**Educational attainment**				
High School	1 (5%)			
Some College	13 (65%)			
Bachelor's degree	6 (30%)			
**Occupation at 9/11**				
Police	15 (75%)			
Other	5 (25%)			
**BMI (kg/m**^**2**^**)**	30.66 ​± ​3.55			
**AUDIT**	2.90 ​± ​1.92			
**Smoking Status**				
Never	18 (90%)			
Former	1 (5%)			
Current	1 (5%)			
**TSPO polymorphism**				
HAB	9 (45%)			
MAB	11 (55%)			
**FEPPA dose (MBq)**	157.14 ​± ​10.77			
**Specific activity (MBq/μg)**	321 ​± ​151.2			
**eTIV (cm**^**3**^**)**	1632.0 ​± ​178.1			

**Note:** Means (±Standard Deviations) or percentages (%) reported.

*Abbreviations:* BMI ​= ​body mass index; AUDIT ​= ​Alcohol Use Disorder Identification Test; HAB ​= ​high affinity binder; MAB ​= ​mixed affinity binder; MBq ​= ​Megabecquerel; eTIV ​= ​estimated total intracranial volume.

### 3.2 TSPO binding and WTC exposures

**Fig. 1:**
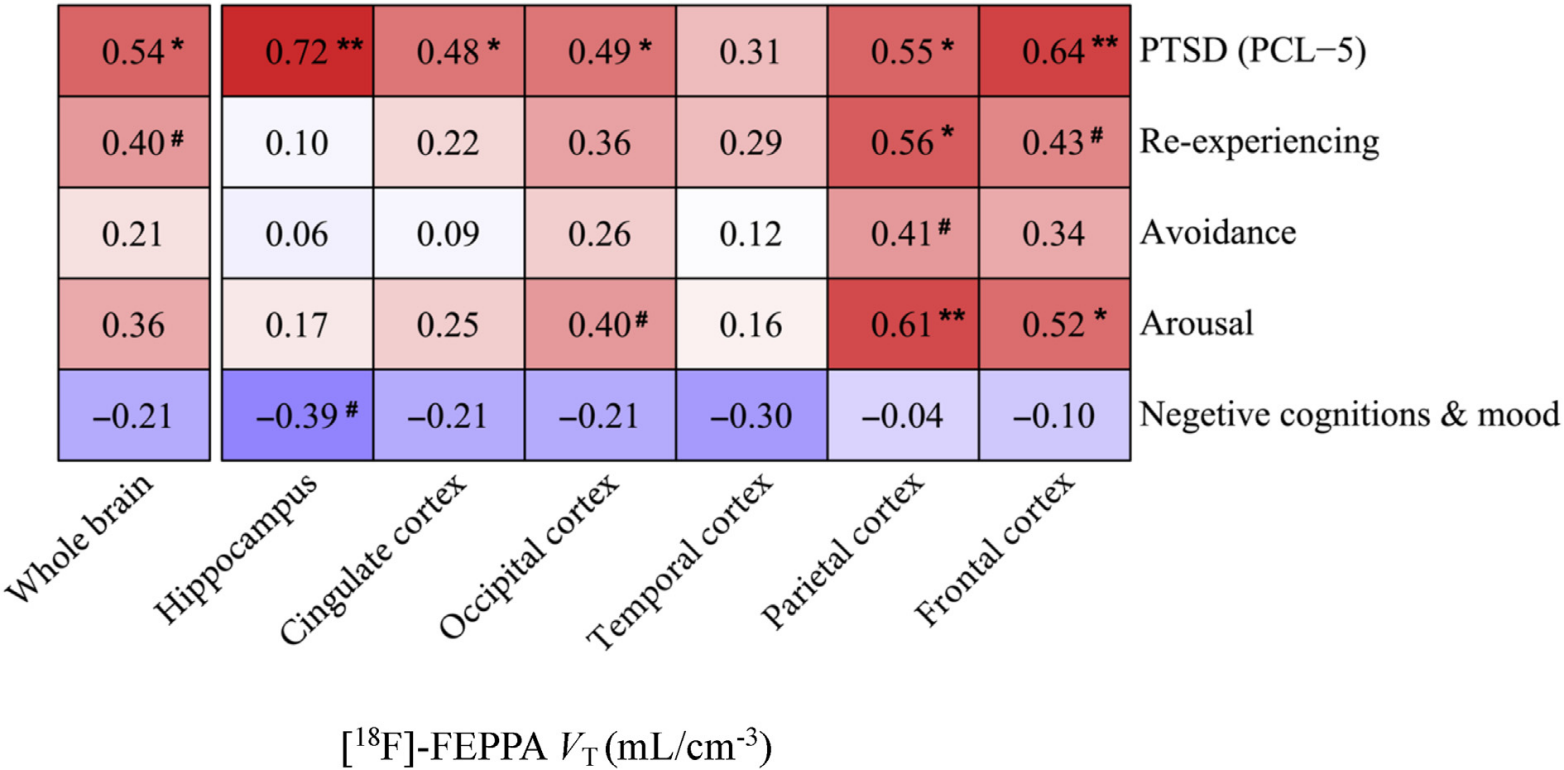
Association between PTSD symptom severity and [^18^F]-FEPPA *V*_T_ adjusted for TSPO *rs6971* genotype in WTC responders **Note:** Regression models showing the relationship between PTSD symptom severity measured as PTSD symptom severity measured as PCL-5 total score and [^18^F]-FEPPA *V*_T_ in the frontal cortex, temporal cortex, parietal cortex, occipital prefrontal cortex, hippocampus, and whole brain adjusted for TSPO *rs6971* genotype (blue circles represent the HABs and red circles represent the MAB). *Abbreviations: V*_T_ ​= ​total distribution volume, CU = WTC responders with unimpaired cognition, MCI ​= ​WTC responders with mild cognitive impairment, HAB ​= ​high affinity binder, MAB ​= ​mixed affinity binder. . (For interpretation of the references to colour in this figure legend, the reader is referred to the Web version of this article.)

Higher global and regional [^18^F]-FEPPA *V*_T_ were significantly associated with greater severity of PTSD symptoms measured by total scores on the PCL-5 (Fig. 1). The strongest associations between [^18^F]-FEPPA *V*_T_ and PTSD symptom severity were found in the hippocampus (*d* ​= ​0.72, r ​= ​0.33, *P* ​= ​0.001) and frontal cortex (*d* ​= ​0.64, r ​= ​0.30, *P* ​= ​0.004). Associations were also found in parietal cortex (*d* ​= ​0.55, r ​= ​0.26, *P* ​= ​0.015), occipital cortex (*d* ​= ​0.49, r ​= ​0.23, *P* ​= ​0.029) and cingulate cortex *(d* ​= ​0.48, r ​= ​0.23, *P* ​= ​0.035), while no association was found in temporal cortex (*d* ​= ​0.31, r ​= ​0.15, *P* ​= ​0.162). All associations remained significant after correcting for multiple comparisons (frontal cortex: *P*_FDR_ ​= ​0.015, parietal cortex: *P*_FDR_ ​= ​0.029, occipital cortex: *P*_FDR_ ​= ​0.040, cingulate cortex: *P*_FDR_ ​= ​0.041, hippocampus: *P*_FDR_ ​= ​0.009 and whole brain: *P*_FDR_ ​= ​0.029).

**Fig. 2:**
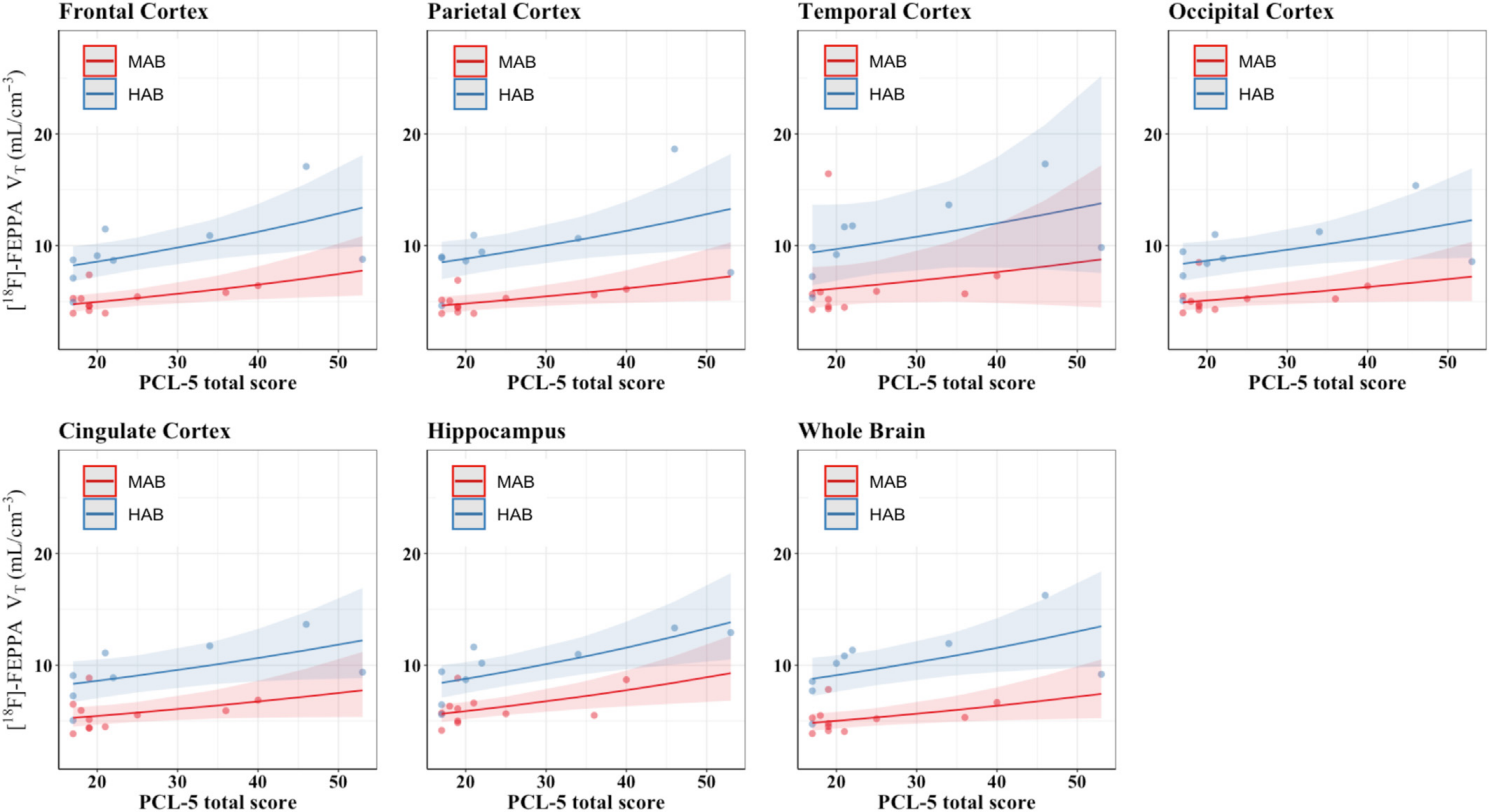
Heat map showing levels of association between dimensional measures of posttraumatic stress disorder and whole brain and regional measures of [^18^F]-FEPPA *V*_T_ in WTC responders. **Note:** Standardized Mean Differences (Cohen's *d*) were estimated from GLM adjusted for TSPO *rs6971* genotype; Standardized Mean Differences deemed statistically significant were noted using the asterisk symbol (∗*P* ​< ​0.05, ∗∗*P* ​< ​0.01). Standardized Mean Differences approaching statistical significance were also noted using the number symbol (^**#**^0.1 ​> ​*P* ​> ​0.05). Red filling indicates higher *V*_T_ level was associated with worse outcome while purple filling indicates lower *V*_T_ level was associated with worse outcome. *Abbreviations: V*_T_ ​= ​total distribution volume, *P* ​= ​nominal p-value.. (For interpretation of the references to colour in this figure legend, the reader is referred to the Web version of this article.)

To investigate whether specific symptom clusters may be driving the association with overall PTSD symptom severity, analyses with PTSD symptom clusters were performed, but results did not identify specific subclusters that were at increased risk in these analyses (Fig. 2). Higher [^18^F]-FEPPA *V*_T_ in the parietal cortex significantly associated with longer time spent at the WTC sites (*d* ​= ​0.44, r ​= ​0.21, *P* ​= ​0.050). There was also a trend towards positive associations between the time spent at the WTC sites and [^18^F]-FEPPA *V*_T_ in the frontal and occipital cortices; however, this did not reach statistical significance (frontal cortex: *d* ​= ​0.40, r ​= ​0.19, *P* ​= ​0.072; occipital cortex: *d* ​= ​0.37, r ​= ​0.18, *P* ​= ​0.098).

Sensitivity analysis exploring the independent contributions of WTC exposures revealed an identical pattern of regional associations for both total PTSD severity and time spent at the WTC sites (Table 2). Moreover, after controlling for PTSD symptom severity, greater exposure duration at the WTC sites was associated with higher [^18^F]-FEPPA *V*_T_ in both the parietal (*d* ​= ​0.50, r ​= ​0.24, *P* ​= ​0.027) and frontal (*d* ​= ​0.49, r ​= ​0.23, *P* ​= ​0.029) cortices. All associations between PTSD symptom severity and TSPO binding remained significant after FDR correction for multiple comparisons.

**Table 2:** Associations between [^18^F]-FEPPA binding and WTC exposures.

Region Name		PTSD severity			Total months on WTC site	
	B ​± ​SE	*d*	*P*	B ​± ​SE	*d*	*P*
Frontal cortex	0.014 ​± ​0.004	0.71	*0.001∗∗*	0.030 ​± ​0.014	0.49	*0.029∗*
Parietal cortex	0.012 ​± ​0.005	0.60	*0.007∗∗*	0.032 ​± ​0.015	0.50	*0.027∗*
Temporal cortex	0.011 ​± ​0.007	0.53		*0.140*	0.035 ​± ​0.023	0.41 *0.136*
Occipital cortex	0.011 ​± ​0.005	0.33	*0.018∗*	0.027 ​± ​0.014	0.33	*0.065*^*#*^
Cingulate cortex	0.011 ​± ​0.005	0.51	*0.025∗*	0.025 ​± ​0.015	0.37	*0.106*
Hippocampus	0.014 ​± ​0.004	0.75	*0.001∗∗*	0.017 ​± ​0.013	0.28	*0.208*
Whole brain	0.012 ​± ​0.005	0.58	*0.010∗*	0.026 ​± ​0.015	0.39	*0.080*^*#*^

**Note:** Regression models showing the relationship between WTC exposures (i.e., PTSD symptom severity and total month working on the WTC sites) and [^18^F]-FEPPA *V*_T_ in the frontal cortex, temporal cortex, parietal cortex, occipital prefrontal cortex, hippocampus, and whole brain (not PVC) in WTC responders. GLM resulted beta coefficients (B), standard error for the beta coefficients (SE), Cohen's *d* (*d*) and nominal p-values (*P*) are reported. ^#^0.1 ​> ​*P* ​> ​0.5, ∗*P* ​< ​0.05 and ∗∗*P* ​< ​0.01.

*Abbreviations: V*_T_ ​= ​total distribution volume.

### 3.3 TSPO binding and cognition

While not the central focus of this report, analyses examining associations between cognitive function and measures of TSPO binding in WTC responders (Appendix Fig. 1) revealed significant association between better visual memory and higher regional [^18^F]-FEPPA *V*_T_ in the frontal cortex (*d* ​= ​−0.46, r ​= ​−0.22, *P* ​= ​0.040), occipital cortex (*d* ​= ​−0.47, r ​= ​−0.23, *P* ​= ​0.036) and hippocampus (*d* ​= ​−0.46, r ​= ​−0.22, *P* ​= ​0.039). However, associations did not survive FDR correction for multiple comparisons. No differences were evident between responders with MCI as compared to those without in unadjusted analyses (Appendix Table 1) and in those adjusted for genotype (Appendix Table 2).

### 3.4 Partial volume correction

Although we did not find group differences in volumetric and cortical thickness measures, all regression models were repeated using the PVC [^18^F]-FEPPA *V*_T_ as the outcome variables. Sensitivity analyses using PVC imaging data did not reveal substantial differences in the results reported using non-PVC reported (data not shown).

## 4 Discussion

WTC responders are at increased risk of chronic PTSD (Bromet et al., 2016), and those who were most severely exposed and those with chronic PTSD are presenting at high rates with MCI at midlife (Clouston et al., 2019). In the present study, we hypothesized that glial activation might be elevated in individuals with PTSD and among those with prolonged WTC exposures, and also considered whether this may also explain decrements in cognitive functioning. In this pilot study, we relied on [^18^F]-FEPPA to characterize the global and regional distribution of TSPO binding in the cerebral cortices and the hippocampus of 20 WTC responders. Our results revealed significant associations between PTSD symptom severity and higher [^18^F]-FEPPA binding, predominantly in the hippocampus and frontal cortex, and longer WTC exposure duration was associated with higher TSPO binding in the parietal cortex. We also found that better visual memory was associated with higher region-specific [^18^F]-FEPPA binding in the frontal cortex, occipital cortex, and hippocampus.

We found that higher PTSD symptom severity was associated with global and regional elevations in [^18^F]-FEPPA binding in most ROIs measured with the strongest associations observed in the hippocampus and frontal cortex. This finding corresponds with a long history of results suggesting that there may be increased risk of inflammation in PTSD (Hori and Kim, 2019, Passos et al., 2015), though there is disagreement about the potential for increased *versus* decreased neuroinflammation in PTSD. Indeed, the only other study investigating the possible association between PTSD symptoms severity and prefrontal limbic TSPO binding reported a significant inverse association in individuals diagnosed with PTSD (Bhatt et al., 2020). The authors of that study suggested that the association between lower TSPO binding and higher PTSD severity may reflect reduced activation of a neuroprotective microglial type or progressive neuroimmune suppression because of depletion of neuroprotective microglia. One possible explanation for this finding is that subsyndromal PTSD may evoke neuroprotective glial activation. Future studies are necessary that seek to further examine the relationship between neuroinflammation and PTSD symptomology.

Another important finding of this study is the association observed between longer time spent at the WTC sites and higher TSPO binding in the parietal cortex. While a few studies have previously linked WTC dust exposure with peripheral inflammation in individuals exposed to the WTC disaster site (Kazeros et al., 2015; Nolan et al., 2012; Sunil et al., 2017), to the best of our knowledge, our study is the first to report an association between WTC environmental exposures and inflammation in the central nervous system. These findings are in line with results from several studies that have linked exposure to particulate matter to the accumulation of dementia-related pathology including oxidative stress and inflammation (Block and Calderon-Garciduenas, 2009; Calderon-Garciduenas et al., 2004; Calderon-Garciduenas et al., 2008). Interestingly, previous work from our research group has also linked the length of time spent at the WTC site with both cognitive dysfunction (Clouston et al., 2017; Clouston et al., 2019) and smaller hippocampal volumetric measures (Deri et al., 2021). Taken together, these results suggest that the brain is adversely affected by the length of time spent at the WTC site and that neuroinflammation may be involved in the underlying pathological process. However, because this effect did not survive multiple comparisons correction, it will be essential to follow-up on this analysis beyond this pilot study with a larger sample to confirm or reject this potential finding. Nevertheless, the potential for this glial activation (protective *versus* harmful) is still unclear and warrants further investigation. Sensitivity analysis suggested that the associations between TSPO binding, and time spent at the WTC sites operated independently of PTSD symptom severity.

It is still unclear what may be driving the inflammatory response observed in exposed individuals almost two decades after the WTC disaster. One possibility is that WTC exposures caused not only chronic PTSD symptomology but also chronic systemic inflammation, that secondarily results in chronic neuroinflammation. Another possibility is that responders with higher PTSD severity were also more heavily exposed to environmental toxins which penetrated the brain and resulted in glial activation.

Neuroinflammatory processes are thought to play a crucial role in the onset of MCI and progression of MCI to AD (Chaney et al., 2019). Results from the present study therefore complicate theory proposed by Calsolaro and Edison (Calsolaro and Edison, 2016), who argued that TSPO was an early marker of the dementing process. Our results therefore support a few existing studies showing small increases in global and regional TSPO in the neocortex in AD-related MCI (e.g., from (Bradburn et al., 2019), *d* ​= ​0.75, 95% C.I. ​= ​[0.42–1.07] in the parietal cortex). One source of difference in our study, as compared to the other studies, is that our study recruited individuals with evidence of recent onset of MCI at midlife. It is also important to note that, whereas the Bradburn meta-analysis relied mostly on older amyloid-positive MCI patients, our responders with MCI are relatively young.

Previous work by our group observed smaller hippocampal volume and reduced cortical thickness in WTC responders with worse cognitive impairment consistent with mild dementia (Clouston et al., 2020; Deri et al., 2021), suggesting involvement of neurodegenerative processes in the development of cognitive impairment in WTC responders. Notably, results from the cortical thickness study were reminiscent of parietal-dominant AD, a subtype of AD that predominantly affects younger individuals (Na et al., 2016). In the current study, no observed changes in structural measures were identified in WTC responders with MCI compared to cognitively unimpaired responders, supporting the view that this analysis occurred in early stage of MCI. However, several studies have previously shown a positive association between neuroinflammation and neurodegeneration in a mixed sample of amyloid positive AD and MCI (Hamelin et al., 2016; Kreisl et al., 2013b), potentially suggesting that although glial activation was not detectable at this early stage of MCI, it may be active at later stages in the dementing process in WTC responders. Future research should seek to conduct longitudinal studies to examine the temporal relationships between neuroinflammation and cognitive changes in WTC responders.

### 4.1 Limitations and future directions

While this study represents the first of its kind to measure TSPO activation at midlife in a cohort of WTC responders with early MCI at midlife, it is limited in several ways. First, this study design had a cross-sectional design and a small sample size. Second, limitations including the unique nature of the exposure, a relatively large number of males and the lack of a suitable external non-WTC-exposed control group for this unique cohort. Third, both PTSD and depression are highly comorbid in the WTC responder cohort, which could indicate that either might help to explain the observed associations with TSPO binding. However, in this study we were not powered to determine the independent contribution of both disorders. Additionally, general weaknesses of using TSPO radioligands, as previously raised by other investigators, is the inability for TSPO tracers to differentiate between more anti-inflammatory and more pro-inflammatory glial activation phenotypes which may allow us to differentiate between beneficial and detrimental neuroinflammatory processes (Bradburn et al., 2019) (Knezevic et al., 2018), and to identify the cellular origin (i.e., astrocyte *versus* microglia) contributing to the observed *in vivo* TSPO signal which may change during the disease course (Tournier et al., 2020a). Research has long highlighted the difference between glial responses and macrophagic responses (Devanney et al., 2020), while methodological work has recently highlighted that brain macrophages have differentially express TSPO and, therefore, may not be identified using TSPO ligands (Nerayan et al., 2017, Pannell et al., 2020). Given prior results focusing on monocytic responses in this population, future neuroimaging work should seek methods for monitoring brain macrophagic reesponses. Despite the shortcomings of TSPO radioligands, TSPO PET remains the most widely used tool for assessing neuroinflammation and there is currently no better validated inflammatory radiotracer available (for review, see (Jain et al., 2020)).

## 5 Conclusions

This is the first study to examine glial activation, measured using *in vivo* TSPO PET imaging in WTC responders at midlife, and to examine the relationship between WTC exposures, PTSD, and glial activation. Our findings suggest that increased TSPO binding was associated with PTSD symptom severity in responders with subsyndromal PTSD, and may also be associated with prolonged exposures to the WTC site. Results from this pilot study suggest that more research is warranted to understand the important role of neuroinflammation in highly exposed WTC responders.

## Declaration of competing interest

The authors declare that they have no known competing financial interests or personal relationships that could have appeared to influence the work reported in this paper.
