# Supplementary material for: Neuroinflammation in World Trade Center responders at midlife: A pilot study using [18F]-FEPPA PET imaging
Source: Brain Behav Immun Health. 2021 Jun 30;16:100287. doi: 10.1016/j.bbih.2021.100287 (PMC8474562; doi:10.1016/j.bbih.2021.100287)
Supplement: Multimedia component 1 [file mmc1.docx]

# Supplementary Appendix

**Appendix Figure 1:** Heat map showing levels of association between dimensional measures of cognition and posttraumatic stress disorder and whole brain and regional measures of [^18^F]-FEPPA *V*_T_ in WTC responders.


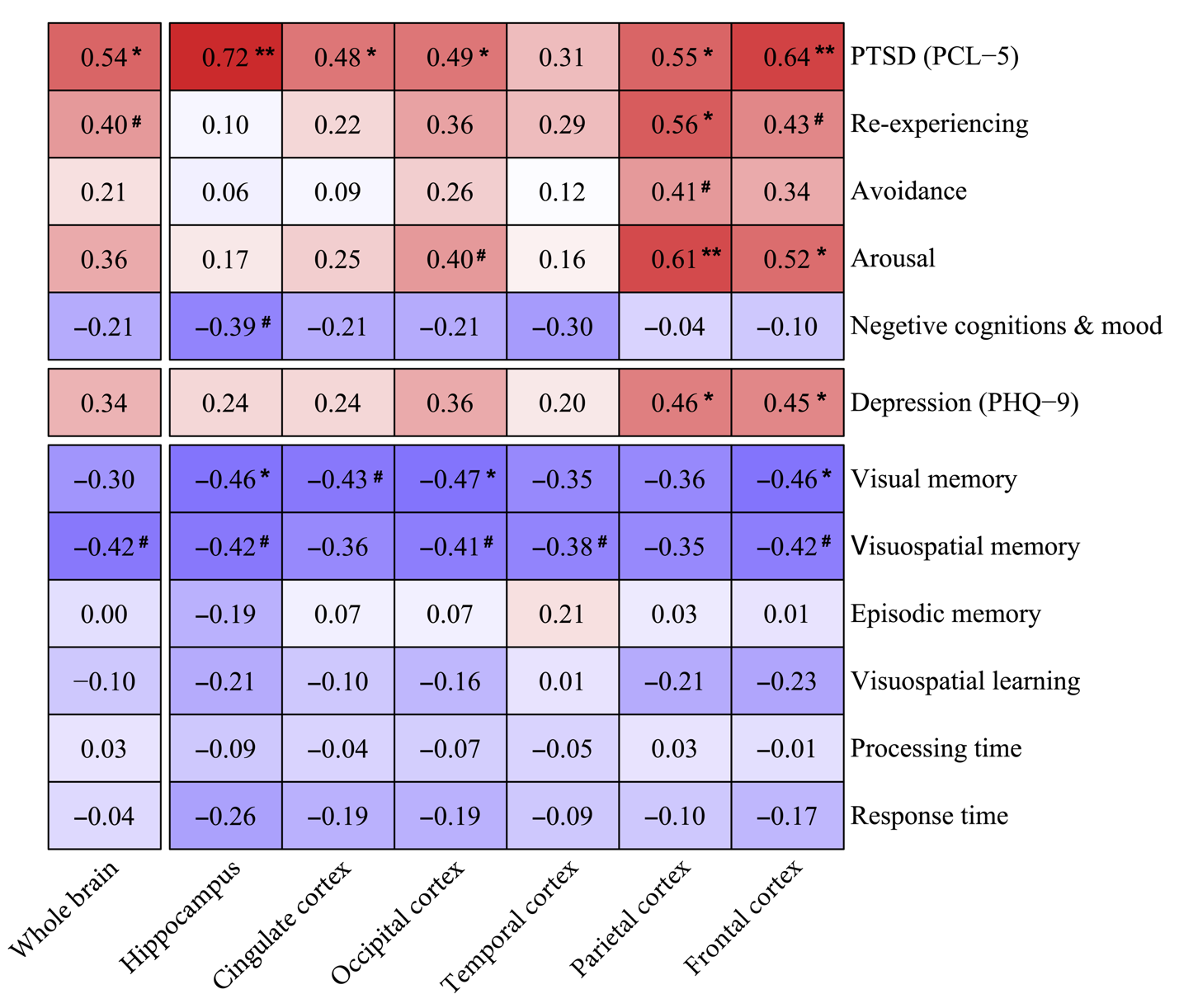


[^18^F]-FEPPA *V*_T_ (mL/cm^-3^)

Neurocognitive measures

**Note:** Standardized Mean Differences (Cohen’s *d*) were estimated from GLM adjusted for TSPO *rs6971* genotype; Standardized Mean Differences deemed statistically significant were noted using the asterisk symbol (**P*<0.05, ***P*<0.01). Standardized Mean Differences approaching statistical significance were also noted using the number symbol (**^#^**0.1>*P*>0.05). Red filling indicates higher *V*_T_ level was associated with worse outcome while purple filling indicates lower *V*_T_ level was associated with worse outcome.

*Abbreviations:* *V*_T_ = total distribution volume, *P* = nominal p-value

**Appendix Table 1:** Global and regional cortical thickness measures (mm^2^) in CU versus MCI

| **Region Name** | **CU** | **MCI** | ***d*** | ***P*** |
| --- | --- | --- | --- | --- |
| **Frontal cortex** | 2.47 ± 0.10 | 2.47 ± 0.08 | *0.03* | *0.941* |
| **Parietal cortex** | 2.32 ± 0.12 | 2.34 ± 0.08 | *0.17* | *0.494* |
| **Temporal cortex** | 2.76 ± 0.10 | 2.75 ± 0.09 | *0.08* | *0.853* |
| **Occipital cortex** | 2.07 ± 0.11 | 2.08 ± 0.06 | *0.14* | *0.762* |
| **Cingulate cortex** | 2.50 ± 0.10 | 2.55 ± 0.10 | *0.47* | *0.353* |
| **Whole brain** | 2.45 ± 0.07 | 2.45 ± 0.10 | *0.07* | *0.885* |

**Note:** Differences in measures of cortical thickness (mm^2^) between groups (CU *versus* MCI) were tested using independent-samples t-test (data represented as mean ± SD). Resulting standardized mean differences (*d*) and nominal p-values (*P*) are reported in the rightmost columns.

Abbreviations: CU = WTC responders with unimpaired cognition, MCI = WTC responders with mild cognitive impairment, *d* = Cohen’s *d*.

**Appendix Table 2:** Global and regional [^18^F]-FEPPA *V*_T_ in MCI and normal cognition subsamples

| Whole brain | Hippocampus | Cingulate cortex | Occipital cortex | Temporal cortex | Parietal cortex | Frontal cortex | Region Name | |
| --- | --- | --- | --- | --- | --- | --- | --- | --- |
| 9.01 ± 3.19 | 9.41 ± 2.70 | 9.24 ± 3.01 | 9.19 ± 2.86 | 10.13 ± 3.55 | 8.80 ± 2.91 | 9.01 ± 2.97 | **HAB** |  |
|  |  |  |  |  |  |  |  | **CU** |
| 5.30 ± 1.62 | 6.47 ± 1.96 | 5.67 ± 1.89 | 5.44 ± 1.74 | 7.16 ± 4.69 | 5.03 ± 1.28 | 5.21 ± 1.45 | **MAB** |  |
|  |  |  |  |  |  |  |  |  |
| 10.94 ± 3.26 | 10.33 ± 2.89 | 9.80 ± 2.73 | 9.70 ± 3.23 | 11.07 ± 3.85 | 10.64 ± 4.53 | 10.15 ± 3.96 | **HAB** |  |
|  |  |  |  |  |  |  |  | **MCI** |
| 5.08 ± 0.42 | 5.74 ± 0.52 | 5.58 ± 0.83 | 5.01 ± 0.35 | 5.34 ± 0.62 | 4.95 ± 0.46 | 5.11 ± 0.51 | **MAB** |  |
|  |  |  |  |  |  |  |  |  |
| 0.06 ± 0.115 | -0.02 ± 0.110 | 0.02 ± 0.116 | -0.02 ± 0.111 | -0.12 ± 0.163 | 0.08 ± 0.118 | 0.04 ± 0.115 | **B ± SE** | |
| 0.12 | -0.05 | 0.03 | -0.04 | -0.17 | 0.15 | 0.08 | ***d*** | |
| *0.581* | *0.820* | *0.896* | *0.850* | *0.454* | *0.515* | *0.715* | ***P*** | |

**Note:** Differences between groups (CU *versus* MCI) are tested using GLM with regional and global *V*_T_ (mL/cm^-3^; mean ± SD) as outcome variables and TSPO *rs6971* genotype (HAB *versus* MAB) as covariate. Resulted beta coefficients (B), standard errors for the beta coefficients (SE), Cohen’s *d* (*d*) and nominal p-values (*P*) are reported.

*Abbreviations:* *V*_T_ = total distribution volume, CU = WTC responders with unimpaired cognition, MCI = WTC responders with mild cognitive impairment.
